# Will the widespread use of large language models in scientific writing undermine scientists’ critical thinking?

**DOI:** 10.1371/journal.pbio.3003801

**Published:** 2026-06-03

**Authors:** Lucas M. Bietti, Adrian Bangerter

**Affiliations:** 1 Department of Psychology, Norwegian University of Science and Technology, Trondheim, Norway; 2 Department of Innovation, WSB Merito University, Warsaw, Poland; 3 Institute of Work and Organizational Psychology, University of Neuchâtel, Neuchâtel, Switzerland

## Abstract

The use of large language models is rapidly transforming the scientific writing process, making it quicker and easier to write research papers. However, this Perspective urges caution when using such tools, arguing that it can risk decoupling writing from thinking.

## Teaser

Artificial intelligence (AI) is rapidly transforming scientific writing by expanding access and efficiency, yet it risks decoupling writing from thinking. Scientific writing is a core cognitive and epistemic practice that must be cultivated and preserved alongside AI use.

Large language models (LLMs) have become deeply embedded in writing practices across society. In science, they herald a profound transformation in how knowledge is produced and communicated. As artificial intelligence (AI)-generated and human writing become increasingly indistinguishable the very notion of authorship, and scientific writing as a distinctly human endeavor, may be reaching its end. In light of this, we would contend that scientific writing is not merely communication but a form of thinking [1]. From this perspective, the increasing outsourcing of writing to AI has two major risks: it alters the signaling function of scientific texts by reducing the cost of producing them [2], and it threatens the development and exercise of core cognitive skills involved in scientific reasoning [3].

LLMs are now used at every stage of the scientific process, from summarizing prior literature to drafting and editing manuscripts, writing grant proposals, and even conducting peer review. Their infiltration of the publishing ecosystem has been swift and largely unregulated. A recent study [4] estimated that more than one in 10 biomedical papers published in 2024 contained AI-generated text, often without disclosure. Another, published in 2025, estimated rates of over 20% in some fields [5]. New detection systems have been developed to identify LLM-generated writing and reviews [6], but they face significant limitations. As a result, the debate has broadened from policing AI use to establishing ethical norms of disclosure and accountability [7].

At the same time, LLMs bring clear benefits. They can support non-native English speakers by reducing linguistic barriers, accelerate literature synthesis, and assist in structuring arguments [8,9]. In educational contexts, they may scaffold learning when used appropriately, for example, by providing feedback, suggesting revisions, or helping students engage with unfamiliar formats [10]. Thus, AI is not inherently detrimental to scientific writing. Its impact depends on how it is integrated into cognitive and epistemic practices.

So, what do we lose when scientific writing gets taken over by AI? Professional writers often gripe about how difficult writing is [10]. Likewise, some scientists believe that “the data should speak for themselves,” viewing writing as post hoc “storytelling” that is sometimes delegated to ghostwriters (who are not even acknowledged as authors). University students, starting with undergraduates all the way up to PhD students, struggle to learn to write effectively, with plagiarism a chronic temptation for those who fail to see the point in making the effort. Perhaps it is a good thing if AI takes over this chore?

We think not. Good scientific writing, and good writing more generally, is hard because, as per the adage, writing is thinking. For humans, writing has been thinking for thousands of years. The emergence of writing fundamentally transformed human cognition by externalizing memory and enabling the storage and transmission of complex information across generations. With writing, thought processes became increasingly dependent on external symbolic systems [11]. This shift reorganized the reliance of the human brain on oral narrative, fostering new forms of analytical, abstract, and self-reflective thinking.

Scientific writing, in particular, promotes clear and critical thinking through the logical organization of ideas and evidence-based argumentation. It also promotes reflection by making implicit insights explicit and sharpening reasoning. Writing enhances comprehension, interest, and self-reflective thinking by engaging cognitive resources that support deeper learning, even in second-language contexts. Furthermore, the effort inherent to the production of a well-written paper can be viewed as an honest signal of quality. In scientific publishing, access to publication is policed by gatekeepers who attend to signals of quality [12–14] such as authors’ identities and affiliations (rightly or wrongly), as well as the quality of the research itself, expressed in part by writing (that is, the thinking). Lower-quality work may manifest in superficial literature integration, poorly motivated hypotheses, inconsistencies between methods and conclusions, or overgeneralized claims not supported by data. AI systems can mask some of these weaknesses by improving fluency and coherence.

Of course, writing is not a perfect signal of quality: many non-native speakers of English face significant discrimination because of their writing, irrespective of the quality of their research or even how good the writing in their papers objectively is. For them, LLMs may help increase access to the publishing market. AI can improve fairness and inclusivity, particularly when used as a tool for linguistic support rather than a cognitive substitute [8].

Nonetheless, the advent of LLMs threatens the signaling equilibrium by lowering the costs of producing good writing. As a result, it becomes easier (cheaper) to produce papers, lowering the threshold for lower-quality papers to enter the market, as evidenced by the increase in AI use in published articles [4,5]. AI-generated papers may be appearing *en masse* on preprint servers as well. Perhaps using AI to review submissions may help offset this trend? The fact that some researchers now insert digital information into the electronic files of submitted manuscripts as secret messages to a purportedly AI reviewer suggests that they now anticipate that their papers may be reviewed by AI. This kind of cat-and-mouse game suggests we may be on the cusp of an arms race between cheaters and regulators of AI in scientific writing. Signaling theory teaches us that, as the value of a signal decreases, communicators learn to ignore the signal. Depending on who wins the arms race, scientific writing may cease to signal quality in research.

Is this really a problem? Egregious cheating may currently be rare, and most scientists probably use AI in good faith (i.e., in ways that are allowed by current guidelines while retaining control of and responsibility for their papers). This includes the use of LLMs to support, rather than replace, core cognitive processes, such as for editing, translation, or brainstorming, while maintaining human responsibility for reasoning, interpretation, and argumentation. However, if enough cheaters invade the market (and it is on average much easier for them to produce papers than for someone relying entirely on their own brainpower and effort), good-faith users of AI in writing will find themselves pressured to increase their reliance on it. This may or may not lead to the collapse of the signaling system, but it will certainly change the way scientists go about the business of scientific thinking. Now consider the fact that the pipeline producing the next generation of scientists is probably compromised in a similar fashion. Students in primary, secondary, and tertiary education are massively using generative AI to accomplish writing assignments. The effects of this shift remain uncertain: some studies suggest benefits for learning and productivity, while others indicate potential negative impacts on critical thinking and retention [10]. We need to understand whether and how this shift may atrophy the next generation of scientists’ ability to think and reason, and we need to be worried about how this will affect scientific writing and (remember, writing is thinking) reasoning in the future. One dystopian outcome of this runaway process may be the degeneration of scientific publications into a subgenre of the AI slop that is currently threatening online culture worldwide.

Science is one of the pinnacles of human culture and writing is its main means of expression. Scientific writing is not only a vehicle for reporting outcomes once the “real” science is done, but a constitutive element of scientific thinking and practice, shaping how ideas are generated and how research is conducted, evaluated, and made publicly accountable. We need to maintain the integrity of scientific writing and continue to cultivate it. If writing is a core cognitive practice through which scientists think, signal quality, and make knowledge accountable, then the unchecked outsourcing of scientific writing to AI risks eroding not only authorship but also the epistemic foundations of science itself.

## Abbreviations

AI: artificial intelligence
LLMs: large language models
